# Comparative Phytochemical Analysis and Antimicrobial Properties of Ethanol and Macerated Extracts from Aerial and Root Parts of *Achillea nobilis*

**DOI:** 10.3390/molecules30142957

**Published:** 2025-07-14

**Authors:** Aiman Berdgaleeva, Zere Zhalimova, Akzharkyn Saginbazarova, Gulbanu Tulegenova, Dana Zharylkassynova, Aliya Bazargaliyeva, Zhaidargul Kuanbay, Svetlana Sakhanova, Akmaral Ramazanova, Akzhamal Bilkenova, Aigul Sartayeva

**Affiliations:** 1 Department of Pharmaceutical Disciplines, Marat Ospanov West Kazakhstan Medical University, 66 Maresyev Street, Aktobe 030019, Kazakhstan; zere_zhalimova@mail.ru (Z.Z.); akzharkyn.sab@mail.ru (A.S.); gul_tulegen@mail.ru (G.T.); zharylkasynova1999@mail.ru (D.Z.); 2 Department of Biology, Natural Sciences Faculty, K.Zhubanov Aktobe Regional University, 34 Moldagulova Avenue, Aktobe 030012, Kazakhstan; aliya_baz@inbox.ru (A.B.); zkuanbay@zhubanov.edu.kz (Z.K.); 3 Scientific and Practical Center, Marat Ospanov West Kazakhstan Medical University, 74 Maresyev Street, Aktobe 030019, Kazakhstan; ssk1968@mail.ru; 4 Department of Neurology with the Course of Psychiatry and Narcology, Marat Ospanov West Kazakhstan Medical University, 66 Maresyev Street, Aktobe 030019, Kazakhstan; akmaral09@mail.com; 5 Department of Natural Sciences, Marat Ospanov West Kazakhstan Medical University, 66 Maresyev Street, Aktobe 030019, Kazakhstan; zhamalok@mail.ru; 6 Department of General Medical Practice No. 2, Marat Ospanov West Kazakhstan Medical University, 66 Maresyev Street, Aktobe 030019, Kazakhstan; sartaeva.73@mail.ru

**Keywords:** *Achillea nobilis*, GC–MS, vortex-assisted extraction, ethanol extract, aerial and root parts, phytochemical profiling, bioactive compounds, traditional medicine, secondary metabolites, aerial oil extract

## Abstract

*Achillea nobilis* represents a species of considerable medicinal importance within the Asteraceae family, historically employed in Central Asia and various Eurasian territories for the management of inflammatory, microbial, and gastrointestinal ailments. Notwithstanding its extensive ethnopharmacological significance, the phytochemical profile and pharmacological attributes of its various anatomical components have not been comprehensively investigated. This research endeavor sought to delineate the phytochemical constituents and evaluate the antimicrobial efficacy of ethanol extracts derived from both the aerial and root segments of *A. nobilis*. Qualitative phytochemical analysis and GC–MS characterization unveiled a diverse array of bioactive compounds, encompassing flavonoids, phenolic compounds, organic acids, lactones, alcohols, and heterocyclic derivatives. In particular, the aerial oil extract exhibited the presence of terpenoids, fatty acids and their esters, sterols, hydrocarbons, and minor organosilicon and cyclobutanone derivatives, with notable compounds such as linoleic acid (8.08%), 6-tetradecyne (14.99%), isopropyl linoleate (14.64%), and *E*,*Z*-1,3,12-nonadecatriene (22.25%). In vitro antimicrobial activity was assessed against eight clinically relevant microbial strains employing the broth microdilution technique. The aerial ethanol extract exhibited pronounced antimicrobial properties, particularly against MRSA and *C. albicans*, with MICs ranging from 0.5 to 2 mg/mL, whereas the root ethanol extract displayed MICs of 1 to 3 mg/mL. Additionally, the aerial oil extract showed moderate inhibitory activity, with MIC values ranging from 1.5 to 3 mg/mL, demonstrating effectiveness particularly against *C. albicans*, *C. neoformans*, and MRSA. These findings underscore the therapeutic potential of *A. nobilis*, particularly its aerial component, as a viable natural source of antimicrobial agents.

## 1. Introduction

**Figure 1:**
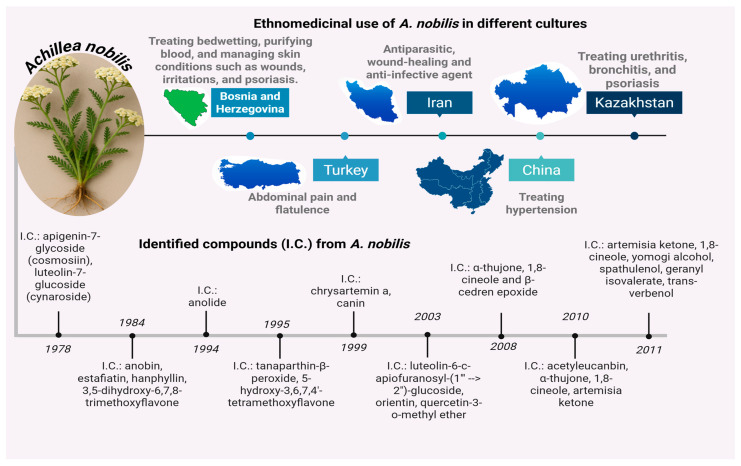
Ethnomedicinal applications and phytochemical discoveries of *A. nobilis* across cultures and time [1,5,6,11,12].

The focused inquiry into flora possessing ethnopharmacological significance persists as an essential avenue for the identification of novel bioactive compounds exhibiting therapeutic potential [1]. *Achillea nobilis*, in conjunction with its subspecies (*A. nobilis* subsp. *sipylea*, *A. nobilis* subsp. *neilreichii*, and *A. nobilis* subsp. *nobilis*), forms a taxonomic assemblage of aromatic medicinal plants that have been historically acknowledged for their varied healing properties throughout Eurasian territories [2,3,4]. Members of this assemblage, classified within the Asteraceae family, have been deeply entrenched in numerous traditional medicinal practices, utilized in the form of decoctions, infusions, essential oils, and topical applications. These formulations have been primarily applied to address a broad range of ailments, encompassing inflammatory conditions, pain syndromes, gastrointestinal disturbances, respiratory infections, and dermatological issues [5,6,7,8,9,10]. The persistent application of *A. nobilis* in ethnomedicine is particularly notable within the healing customs of nations such as Bosnia and Herzegovina, Turkey, Iran, China, and Kazakhstan, where the plant is imbued with both cultural and pharmacological importance (Figure 1) [1,2,11].

*A. nobilis*, or “noble yarrow,” is a perennial herbaceous species of the Asteraceae family, typically growing 15–70 cm tall. It features hairy, longitudinally ridged stems and woolly-pubescent, pinnately divided leaves that vary between basal and upper parts. The plant forms dense, corymbose inflorescences with numerous small capitula containing pale yellow and white florets. Flowering occurs from June to August, and the species is well-adapted to temperate Eurasian habitats [1,2,3].

Between the years 1978 and 2011, phytochemical investigations concerning *A. nobilis* predominantly concentrated on the aerial segments of the organism, with insufficient focus directed towards its subterranean roots and lipid-soluble constituents [12]. The most commonly employed methodology for extraction was hydrodistillation, particularly for the purpose of isolating volatile compounds from the flowering aerial tissues [13,14,15,16,17,18,19]. Initial studies employed chromatographic techniques in conjunction with ultraviolet and infrared spectroscopy to identify flavonoid glycosides such as apigenin-7-glycoside (cosmosiin) and luteolin-7-glucoside (cynaroside) [17,18]. Subsequent investigations integrated advanced methodologies, including infrared spectroscopy, nuclear magnetic resonance, thin-layer chromatography, ultraviolet spectroscopy, gas chromatography–mass spectrometry, electrospray ionization mass spectrometry, and high-performance liquid chromatography, to further elucidate the plant’s secondary metabolites [16,17,18]. Through the application of these methodologies, a diverse array of chemical classes were discerned, encompassing sesquiterpene lactones, methoxylated flavones, dihydroxyflavones, and peroxide derivatives. Compounds such as anobin, estafiatin, hanphyllin, tanaparthin-*β*-peroxide, and chrysartemin A were successfully isolated from chloroform and ethanol extracts, while 1,8-cineole, camphor, *α*-thujone, *β*-caryophyllene, and artemisia ketone were identified in hexane and dichloromethane fractions utilizing gas chromatography–mass spectrometry [20,21,22,23]. Despite some investigations also employing liquid–liquid partitioning and solid-phase microextraction techniques, the chemical profiling of non-polar fractions and subterranean components has remained largely unexplored. The persistent emphasis on aerial parts and polar solvent systems underscores a significant gap in the phytochemical characterization of *A. nobilis*, thereby accentuating the imperative for future research endeavors aimed at investigating root-derived and lipid-soluble constituents to comprehensively assess the plant’s medicinal potential.

Pharmacological research on *A. nobilis* has shown a variety of bioactivities that back its traditional applications and healing potential. Importantly, ethanolic extracts of *A. nobilis* showed considerable anticonvulsant effects in animal seizure models, such as maximal electroshock (MES), pentylenetetrazole (PTZ), and strychnine nitrate (STN)-induced seizures. The effects were dependent on the dosage, with 60% protection noted at 300 mg/kg in the STN model, indicating a role in glycine-mediated neurotransmission [7,24]. Additionally, *A. nobilis* subsp. *neilreichii* showed antinociceptive and anti-inflammatory properties in rodent studies, particularly alleviating inflammatory pain in formalin and carrageenan-induced paw edema tests, although it was less efficient in thermal nociception trials [8,25]. *A. nobilis* subsp. *sipylea* demonstrated antispasmodic effects in isolated rat duodenum samples, functioning through calcium channel modulation instead of muscarinic antagonism, suggesting smooth muscle relaxation capabilities [7,8,22,23]. Research on antioxidants showed that *A. nobilis* infusions increased catalase and glutathione peroxidase activities in erythrocytes and leucocytes, offering protection against oxidative damage caused by hydrogen peroxide [7,23,24].

The antimicrobial efficacy of *A. nobilis* and its various subspecies has been corroborated by numerous investigations, which illustrate inhibitory effects against a wide array of Gram-positive and Gram-negative microorganisms. In a comparative analysis, essential oils derived from *A. nobilis* subsp. *sipylea* and *A. nobilis* subsp. *neilreichii* displayed significant activity against pathogens such as *Escherichia coli*, *Staphylococcus aureus*, *Salmonella typhimurium*, and *Candida albicans*, with inhibition zones ranging from 9 to 41 mm contingent upon the specific bacterial strain and the provenance of the plant material [9]. Furthermore, when assessed alongside conventional antibiotics such as amoxicillin and sulbactam/ampicillin, the essential oils demonstrated comparable effectiveness in certain instances, particularly against *S. aureus* and *P. vulgaris* [9]. Additional investigations revealed that *A. nobilis* essential oil accomplished inhibition zones of 25 mm against *E. coli*, *Klebsiella pneumoniae*, and *S. aureus*, and 23 mm against *Pseudomonas aeruginosa*, thereby surpassing *A. crithmifolia* and even thymol in specific scenarios [15]. A comprehensive screening employing the disc diffusion methodology indicated that *A. nobilis* subsp. *neilreichii* essential oil inhibited various strains, including *Bacillus megaterium*, *Listeria monocytogenes*, and *K. pneumoniae*, with inhibition zones ranging from 3 to 13 mm [21]. These outcomes bolster the proposition that the antimicrobial effects are contingent upon species and strain, likely influenced by the phytochemical constituents of the essential oil. Moreover, the choice of solvent and extraction method significantly influences the antimicrobial potency, as illustrated in antioxidant studies which demonstrated that ethanol and ethanol–water extracts exhibited superior radical scavenging and reducing capabilities, potentially correlating with antimicrobial efficacy [20].

Our research aims to provide a comprehensive analysis of the phytochemical composition of *A. nobilis* by examining both the aerial and root parts using two complementary extraction approaches: ethanol extraction aided by vortexing and maceration-based oil extraction. Subsequently, GC–MS profiling was performed to identify a broad spectrum of bioactive compounds potentially responsible for the plant’s traditional medicinal applications. Previous studies have largely focused on hydrodistilled or aqueous–alcoholic extracts from the aerial parts, often neglecting the chemical diversity of underground organs and lipid-soluble fractions. To overcome this limitation, we implemented vortex-assisted ethanol extraction, which enhances solvent penetration and facilitates efficient recovery of both polar and moderately non-polar constituents. In parallel, macerated oil extraction of the aerial part was conducted to selectively isolate lipophilic compounds, such as fatty acids, terpenoids, sterols, and hydrocarbons, which are not readily extracted with polar solvents. The inclusion of oil-based extraction was further motivated by its practical relevance for topical formulations. Given the traditional external use of *A. nobilis* in folk medicine, the oil extract serves as a prototype for the development of therapeutic ointments and dermal applications, where lipid-soluble phytoconstituents can exert antimicrobial, anti-inflammatory, or antioxidant effects directly at the site of application. This dual-extraction strategy not only expands the phytochemical profile of the species but also provides a pharmacologically meaningful foundation for the development of evidence-based phytotherapeutic products targeting both systemic and topical uses.

## 2. Results

### 2.1. Screening for Bioactive Phytochemical Classes

Phytochemicals consist of a wide variety of naturally found plant substances, numerous of which are linked to medicinal attributes. A qualitative phytochemical analysis was performed to acquire an initial insight into the medicinal potential of *A. nobilis*. As outlined in Table 1, extracts from both the aerial and root sections of the plant, as well as the aerial oil extract, demonstrated the existence of several important phytochemical categories. The screening validated the presence of biologically active components including alcohols, aldehydes, amines, amides, alkaloids, triterpenoids, tannins, flavonoids, and glycosides.

In the aerial oil extract, alcohols, aldehydes, and triterpenoids were prominently detected, while flavonoids and glycosides were also present in moderate amounts. In contrast, amines, amides, tannins, and alkaloids were not detected in the oil-based extract.

### 2.2. Secondary Metabolite Analysis (GC–MS)

**Figure 2:**
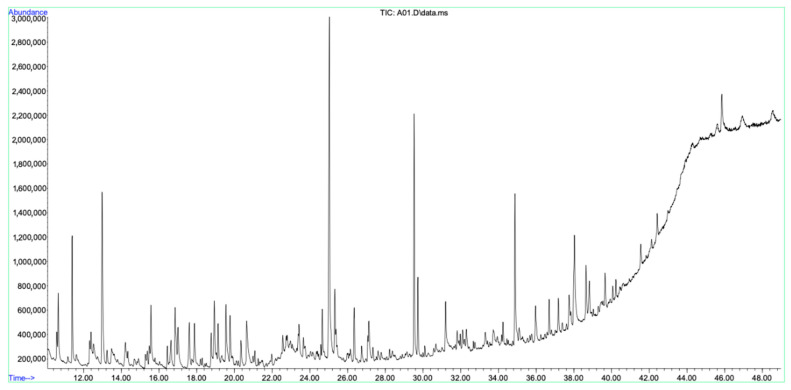
GC–MS chromatogram of aerial ethanol extract constituents of *A. nobilis*.

**Figure 3:**
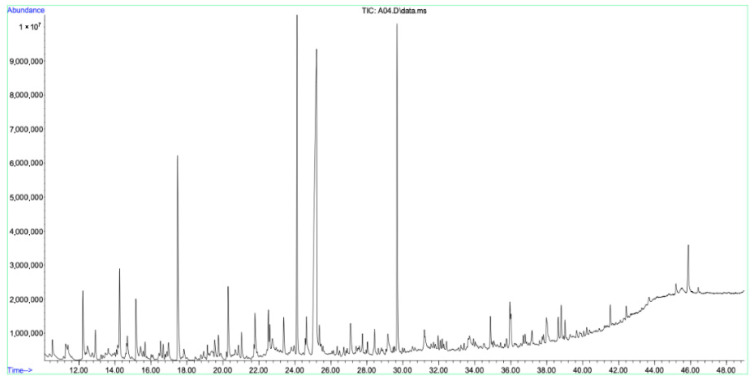
GC–MS chromatogram of the root ethanol extract of *A. nobilis.*

An extensive GC–MS evaluation of the aerial ethanol extract, root ethanol extract, and aerial oil extract of *A. nobilis* showed a wide variety of phytochemical compounds. Ethanol was chosen as the extraction solvent because of its wide-ranging ability to dissolve various polar to moderately non-polar bioactive substances and its appropriateness for subsequent biological and pharmacological assessments. A total of 38 compounds were recognized in the aerial ethanol extract (Table 2, Figure 2), while 58 compounds were found in the root ethanol extract (Table 3, Figure 3). Of these, 22 compounds were found in both plant sections, indicating a level of phytochemical similarity. These common constituents were additionally categorized according to their relative concentrations into three specific groups: minor compounds (present at concentrations <1%), common compounds (*n* = 22), and major compounds (concentrations ≥1%).

**Figure 4:**
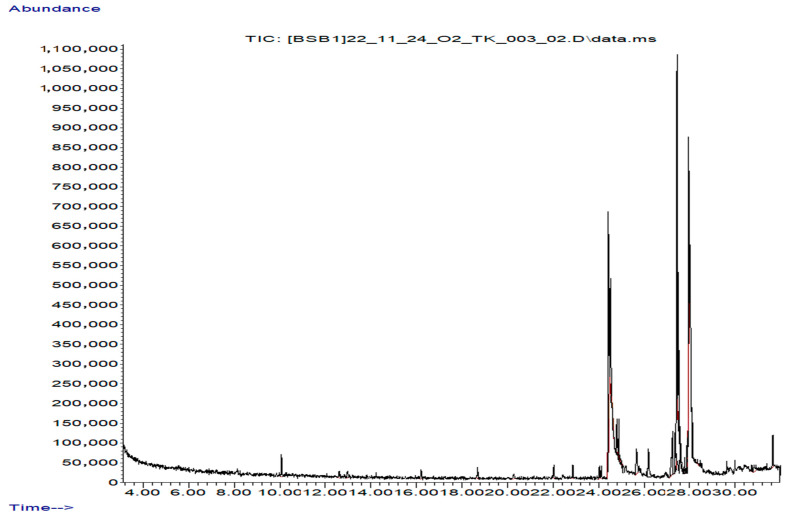
GC–MS chromatogram of the aerial oil extract of *A. nobilis.*

Furthermore, 25 compounds were identified in the aerial oil extract (Table 4, Figure 4), of which 9 were minor compounds (present at concentrations <1%) and 16 were major compounds (≥1%). These compounds belonged to diverse phytochemical classes including terpenoids, heterocyclic compounds, fatty acids, fatty acid esters, hydrocarbons, amides, vitamins, cyclobutanone derivatives, organosilicon compounds, and sterols. Compared to the aerial ethanol extract and root ethanol extract, the aerial oil extract exhibited a more selective enrichment of lipophilic bioactives, especially terpenoid and fatty acid derivatives, consistent with the solvent’s affinity for non-polar constituents.

In the aerial ethanol extract, the compounds identified at concentrations under 1% included R-(–)-1,2-propanediol (0.89%), *γ*-butyrolactone (0.95%), N-nitrosohexamethyleneimine (0.19%), 1,2-cyclopentanedione (0.54%), and 5-tert-butylpyrogallol (0.60%). In comparison, the root ethanol extract displayed a broader spectrum of minor elements, including but not restricted to 4-cyclopentene-1,3-dione (0.81%), 2(5H)-furanone, 3-methyl- (0.39%), 2(5H)-furanone (0.97%), 1,2-cyclopentanedione, 3-methyl- (0.69%), and 2-cyclopenten-1-one, 3-ethyl-2-hydroxy- (0.54%). Other minor components included ethanone, 1-(1H-pyrrol-2-yl)- (0.45%), 2(3H)-furanone, 5-heptyldihydro- (0.70%), allyl acetate (0.85%), 1,2,3-propanetriol, 1-acetate (0.42%), acetaminophen (0.09%), and uric acid (0.70%). Trace amounts of various nitrogen-containing heterocycles and phenolic derivatives were also detected, including 2-aminopyrimidine-1-oxide (0.53%), 3-pyridinol, 6-methyl- (0.32%), and uracil (0.56%).

The aerial ethanol extract comprised a collection of significant compounds, such as 2,3-butanediol (6.08%), butanoic acid (1.89%), (L)-*α*-terpineol (1.53%), methyl N-hydroxybenzenecarboximidate (2.69%), and ethanol, 2,2’-oxybis- (1.82%). Other notable components included phenol, 4-ethyl-2-methoxy- (1.08%), 1,3-propanediol (2.20%), eugenol (1.49%), and dianhydromannitol (1.07%). Significantly, 3-isobutylhexahydropyrrolo [1,2-a]pyrazine-1,4-dione (1.27%) and octadecanoic acid (1.42%) also played a major role in shaping the extract’s chemical profile. In contrast, the root ethanol extract demonstrated a wider range of chemical variety among its primary components. Significant compounds included 2-propenoic acid (1.16%), 2-furanmethanol (1.14%), 2,4-dimethyl-2-oxazoline-4-methanol (3.01%), and 1,2-cyclopentanedione (2.55%). The compound present in the highest quantity was benzaldehyde, 3-hydroxy-, oxime (6.65%). Other notable compounds included cyclopropyl carbinol (1.06%), 1,3-dioxol-2-one, 4,5-dimethyl- (1.39%), *α*-hydroxy-*γ*-butyrolactone (1.26%), and 4H-pyran-4-one, 2,3-dihydro-3,5-dihydroxy-6-methyl- (9.39%). The latter showed the greatest relative abundance among all recognized components. In addition, 3-pyridyl ester of benzoic acid (1.88%) and butyl 9-decenoate (1.58%) were also common.

The aerial oil extract contained a distinct composition with 25 identified compounds. Among these, 9 were minor compounds (present at <1%): camphor (1.41%), 2,4-decadienal (0.46%), 2-furanacetaldehyde, *α*-propyl- (0.69%), 3-(hydroxymethylene)indolin-2-one (0.66%), hedycaryol (0.70%), 6-methyl-2,4(1H,3H)-pteridinedione (0.57%), tridecanoic acid, methyl ester (0.92%), 11,14-octadecadienoic acid, methyl ester (0.68%), 4-methyl-3-pentenal (0.60%), and 1-(trimethylsilyl)-1-propyne (0.49%). The major compounds (≥1%) in the aerial oil extract were hexadecanoic acid, ethyl ester (1.15%), linoleic acid (8.08%), linoleic acid ethyl ester (1.75%), oleic acid ethyl ester (2.28%), palmitoyl chloride (2.50%), *α*-tocopherol (8.36%), *E*,*Z*-1,3,12-nonadecatriene (22.25%), oleic acid, 3-hydroxypropyl ester (6.92%), 2-dodecylcyclobutanone (1.24%), isopropyl linoleate (14.64%), *cis*-13,16-docasadienoic acid (1.42%), chondrillasterol (1.54%), and squalene (2.35%).

The GC–MS analysis showed a wide range of chemical classes present in the aerial ethanol extract, root ethanol extract, and aerial oil extract of *A. nobilis* (Table 5). Both ethanol-based extracts contained high levels of alcohols, carboxylic acids, ketones, phenols, and heterocyclic compounds. Alcohols constituted an important category, including various diols and polyether diols found in both sections of the plant. Carboxylic acids, especially saturated fatty acids and short-chain fatty acids, were prevalent in both extracts, highlighting the metabolic diversity of the plant.

Significantly, the root ethanol extract exhibited a greater presence of distinct chemical classes like carbonate derivatives, sugar lactones, nitrosoamines, and oxazoline derivatives, which were not found in the aerial ethanol extract. In contrast, dioxolones and imidates were only found in the aerial ethanol extract. Additionally, phenolic compounds, often linked to antioxidant and antimicrobial benefits, were found in both extracts, although certain phenol derivatives were exclusive to one section. Overall, the root ethanol extract demonstrated the greatest variety of chemical classes, while the aerial oil extract exhibited selective enrichment in lipid-soluble phytochemicals, contributing additional pharmacologically relevant classes not seen in polar extracts.

The categorization of the detected compounds revealed clear differences in distribution patterns between the aerial and root extracts. The aerial extract of the alcohols mainly included diols and monoterpene alcohols, whereas the root extract presented other subclasses like cyclopropyl alcohols and polyether diols. Among the carboxylic acids, both extracts included short-chain fatty acids and *γ*-hydroxy acids; however, the root ethanol extract specifically had *α,β*-unsaturated acids and hydroxybutyrolactones.

The aerial oil extract contained significant amounts of long-chain unsaturated fatty acids, such as linoleic and oleic acid derivatives, along with their esters. This extract also included tocopherols, triterpenoids, and cyclobutanone derivatives, representing lipid-soluble classes rarely detected in ethanol-based profiles.

Phenolic subclasses also exhibited variation, with methoxyphenols, dimethoxyphenols, and hydroquinone derivatives found in both parts, whereas alkylated methoxyphenols and trihydroxybenzene derivatives were observed solely in the aerial ethanol extract. The root ethanol extract exhibited significant diversity in the heterocyclic class, featuring oxazoline, pyrimidine, and pyridinol derivatives, while the aerial extract contained various pyrrole and imidate derivatives. Furthermore, subclasses like sugar alcohols, dihydroxybenzenes, and pyranones distinguished the two extracts further.

**Figure 5:**
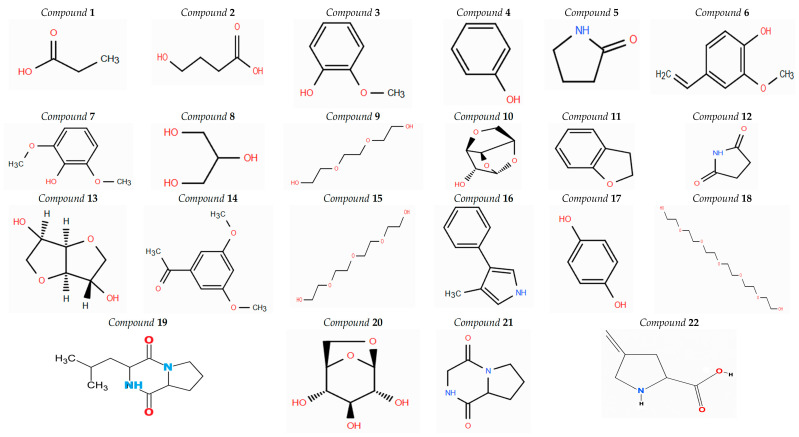

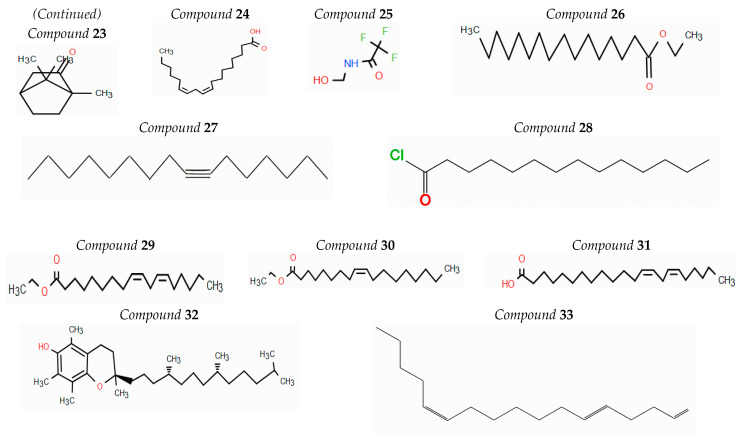

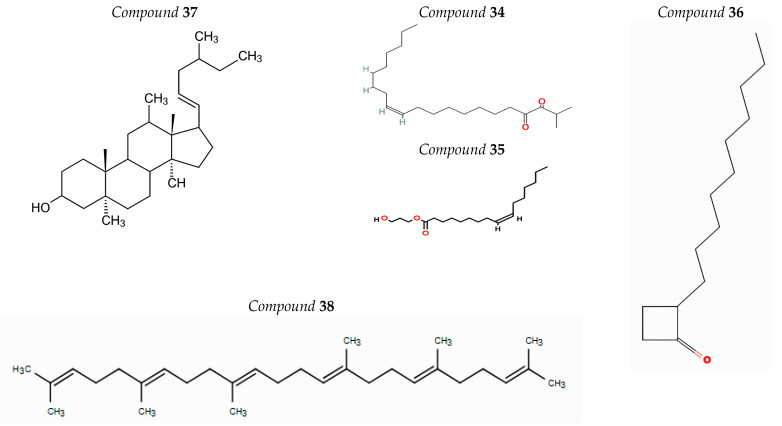
Chemical structures of common compounds identified in the aerial and root extracts of *A. nobilis*. Here, the numbering follows the one used in Table 6.

Of the 22 shared phytochemical components found in both aerial ethanol and root ethanol extracts of *A. nobilis*, several were detected at levels under 1% in both sections of the plant (Figure 5, Table 6). These comprised propanoic acid (0.92% in aerial ethanol extract, 0.96% in root ethanol extract), benzofuran, 2,3-dihydro- (0.60%, 0.41%), succinimide (0.48%, 0.34%), and 3-methyl-4-phenyl-1H-pyrrole (0.61%, 0.48%). Moreover, *β*-D-glucopyranose, 1,6-anhydro- (1.39%, 0.33%) and 1,4:3,6-dianhydro-*α*-D-glucopyranose (1.76%, 0.4%) demonstrated asymmetric occurrence, surpassing 1% solely in the aerial ethanol extract. These minor components, despite being found in comparatively small amounts, can have considerable pharmacological impacts via synergistic processes or act as indicators for particular therapeutic functions.

In the aerial oil extract, additional minor compounds identified at levels under 1% included 2,4-decadienal (0.46%), 2-furanacetaldehyde, *α*-propyl- (0.69%), 3-(hydroxymethylene)indolin-2-one (0.66%), hedycaryol (0.70%), 6-methyl-2,4(1H,3H)-pteridinedione (0.57%), 4-methyl-3-pentenal (0.60%), 11,14-octadecadienoic acid, methyl ester (0.68%), and 1-(trimethylsilyl)-1-propyne (0.49%).

Multiple compounds were consistently found at elevated levels (>1%) in both aerial ethanol and root ethanol extracts, suggesting their prevalence and possible functional significance in *A. nobilis*. 4-hydroxybutanoic acid (7.76% in aerial ethanol extract, 2.34% in root ethanol extract) and glycerin (15.97%, 6.73%) were some of the main components. 2-methoxy-4-vinylphenol, a recognized phenolic substance, was significantly found (3.26%, 1.10%), in addition to phenol, 2,6-dimethoxy- (2.85%, 1.57%). Other frequently occurring shared compounds included triethylene glycol (2.80%, 1.05%), tetraethylene glycol (3.22%, 1.95%), hydroquinone (1.74%, 1.35%), and pyrrolo[1,2-a]pyrazine-1,4-dione, hexahydro-3-(2-methylpropyl)- (2.19%, 1.55%). These findings emphasize that both parts of the plant hold a fundamental set of key bioactive compounds that could greatly enhance the overall medicinal properties of *A. nobilis*. In the aerial oil extract, compounds detected at levels >1% included camphor (1.41%), hexadecanoic acid, ethyl ester (1.15%), linoleic acid ethyl ester (1.75%), oleic acid ethyl ester (2.28%), 2,2,2-trifluoro-N-(hydroxymethyl)acetamide (3.35%), palmitoyl chloride (2.50%), *α*-tocopherol (8.36%), *E,Z*-1,3,12-nonadecatriene (22.25%), oleic acid, 3-hydroxypropyl ester (6.92%), 2-dodecylcyclobutanone (1.24%), isopropyl linoleate (14.64%), *cis*-13,16-docasadienoic acid (1.42%), chondrillasterol (1.54%), and squalene (2.35%).

### 2.3. Determination of Antimicrobial Potency

The expected minimum inhibitory concentrations (MICs) of ethanol extracts from the aerial and root sections of *A. nobilis* against a selection of clinically significant microbial strains are presented in the related data (Table 7). The aerial extract showed enhanced antimicrobial effectiveness overall, exhibiting lower MIC values specifically against *C. albicans* (0.75 mg/mL), MRSA (0.50 mg/mL), and *C. neoformans* (0.85 mg/mL), in contrast to the root extract. Additionally, the aerial oil extract demonstrated considerable antimicrobial effects, with MIC values of 0.9 mg/mL against MRSA, 1.2 mg/mL against *C. albicans*, and 1.4 mg/mL against *C. neoformans*.

Despite the root extract demonstrating less potent inhibitory effects, it still displayed moderate activity against all tested strains, indicating that both parts of the plant have broad-spectrum antimicrobial potential. The aerial oil extract also showed MIC values of 1.5–3.2 mg/mL against Gram-negative bacteria, reinforcing its comparable effectiveness. Importantly, the extracts showed efficacy against Gram-positive and Gram-negative bacteria, along with fungal pathogens, reinforcing the traditional application of *A. nobilis* for treating infections and emphasizing its potential as a source of natural antimicrobial compounds, which may contribute to alternative therapeutic strategies amid rising antibiotic resistance and the need for plant-derived agents with multifunctional bioactivity.

## 3. Discussion

The increasing worldwide fascination with bioactive natural products highlights the essential requirement for thorough scientific research on medicinal plant species. Plants historically utilized in folk medicine for various health issues are now gaining heightened interest from researchers in various fields because of their possible pharmacological properties and enduring ethnomedicinal significance. *A. nobilis* is one such plant, historically esteemed in Central Asia for its medicinal qualities, akin to other species in the *Achillea* genus [7,8,17]. Even with its recorded traditional uses, a considerable gap persists in focused phytochemical studies that specifically target this species. The lack of thorough chemical and pharmacological investigations not only restricts our comprehension of its possible therapeutic advantages but also hinders the identification of new compounds that might aid in creating evidence-based therapies. As a result, an important source of natural therapeutic compounds might be neglected. Thus, prioritizing the phytochemical identification and pharmacological assessment of important regional plants such as *A. nobilis* is crucial to reveal their complete medicinal capabilities and facilitate their incorporation into contemporary healthcare practices.

The preliminary phytochemical screening of *A. nobilis* revealed the presence of various major classes of bioactive compounds. In the aerial ethanol extract, alcohols, aldehydes, amines, flavonoids, triterpenoids, and glycosides were detected. The aerial oil extract tested positive for alcohols, aldehydes, alkaloids, and triterpenoids. Meanwhile, the root ethanol extract contained flavonoids, alkaloids, and glycosides. These findings are consistent with previous studies on various members of the *Achillea* genus, where flavonoids, terpenoids, and phenolic compounds have been widely reported as dominant constituents contributing to their anti-inflammatory, antimicrobial, and antioxidant properties [61,62,63,64]. Compared to species such as *A. millefolium* and *A. wilhelmsii*, which are well-characterized for their high flavonoid and sesquiterpene lactone contents, *A. nobilis* shows a similar phytochemical pattern but demonstrates a broader distribution of classes across both plant organs [64]. Moreover, the presence of glycosides and triterpenoids, particularly in the root extracts, highlights the potential for underexplored pharmacological effects unique to this species. These results emphasize the importance of expanding phytochemical research within the *Achillea* genus to include lesser-studied species like *A. nobilis*, which may serve as reservoirs of novel bioactive metabolites with therapeutic relevance [65,66,67].

The GC–MS analysis of the aerial ethanol extract of *A. nobilis* identified various pharmacologically active compounds that contribute to the plant’s traditional and modern medicinal relevance. Among them, 2,3-butanediol demonstrated a broad range of biological activities, including antitumor and immunomodulatory effects, cryoprotective properties, and utility as a drug carrier, alongside anti-inflammatory, neuroprotective, and antimicrobial potential [26,27]. 1,3-Propanediol was also identified, known for its application in pharmaceutical formulations as a solvent, humectant, and stabilizer [28]. Notably, butanoic acid, a histone deacetylase (HDAC) inhibitor, links to epigenetic modulation and potential anticancer effects [33]. Octadecanoic acid displayed a wide therapeutic range including antimicrobial, anti-inflammatory, antioxidant, anticancer, emollient, cholesterol-lowering, and immunomodulatory properties [34]. Other relevant compounds included dianhydromannitol, known for diuretic, osmoprotective, and excipient-related functions [40], *γ*-butyrolactone, which acts on the central nervous system as a sedative and anxiolytic but warrants caution due to its association with GHB-related dependence [43], and eugenol, a well-studied bioactive with antibacterial, antifungal, antiviral, anticancer, anti-inflammatory, and antioxidant activity [45].

In contrast, the aerial oil extract exhibited a distinct phytochemical profile, rich in lipophilic bioactives. For instance, *α*-tocopherol is a potent antioxidant with additional anti-inflammatory, cytoprotective, neuroprotective, and mitochondrial stabilizing properties; it also modulates signal transduction and lipid metabolism, and protects non-cancerous cells during chemotherapy [57,58,59,60]. Linoleic acid, a major polyunsaturated fatty acid, was detected, with confirmed anti-proliferative, pro-apoptotic, antioxidant, and anti-inflammatory functions, along with EMT and angiogenesis inhibition and immune modulation via mitochondrial biogenesis pathways (PGC-1*α*/NRF1/TFAM) [34,35]. Squalene, a triterpene, is known for its antioxidant, anti-inflammatory, anticancer, and antidiabetic effects [52]. Chondrillasterol demonstrated antimicrobial activity, adding to the therapeutic profile of the oil extract [56]. Another major compound, camphor, has been shown to possess antifungal activity against *Rhizoctonia solani* and other pathogens [53], antiviral action against orthopoxviruses [54], and cytotoxicity toward cancer cells through various mechanistic pathways [55].

The GC–MS examination of the root extract from *A. nobilis* identified various unique bioactive compounds with specific pharmacological significance. Among these, 2-furanmethanol was recognized, a substance known for its antimicrobial, antifungal, and anticancer properties, indicating its possible role in the plant’s conventional medicinal uses [31]. Furthermore, 4-cyclopentene-1,3-dione was exclusively detected in the root extract and is recognized for its significant antifungal characteristics, further reinforcing the antifungal potential of constituents derived from the root [42]. Another notable compound, 2,3-dimethylhydroquinone, showed antioxidant and antimicrobial properties, as well as cytotoxic and redox-modulating actions, suggesting a potential function in managing oxidative stress and pathways linked to cancer [49]. The identification of uric acid is especially significant, as it functions as a biomarker for cardiovascular and metabolic diseases, and contributes to inflammation in conditions like gout due to crystal-induced inflammation in hyperuricemic conditions [50]. Ultimately, uracil was identified and is linked to a wide variety of pharmacological effects, encompassing antiviral, anticancer, and antimicrobial actions, as well as its role in DNA and RNA synthesis, enzyme inhibition, radiosensitization, and immunomodulation [51]. Together, these compounds underscore the therapeutic potential of *A. nobilis* roots, which have been relatively overlooked, and stress the necessity for additional pharmacological and mechanistic studies regarding their effectiveness as treatments.

The GC–MS examination of *A. nobilis* identified a set of bioactive compounds typically found in both aerial and root extracts, all exhibiting a broad range of pharmacological effects. Glycerin acts as a powerful humectant and lubricant, commonly utilized for its ability to retain moisture in pharmaceutical and cosmetic uses [29]. In a similar vein, triethylene glycol demonstrates significant antimicrobial and antiviral properties, as well as disinfectant and air-purifying capabilities, establishing it as a versatile agent with pharmaceutical importance [30]. Propanoic acid offers antimicrobial and anti-inflammatory properties, and additionally influences lipid metabolism regulation, modulates gut microbiota, and inhibits HDAC, thus reinforcing its possible anticancer and immune-modulatory functions [32]. Additionally, 4-hydroxybutanoic acid is recognized for its sedative, muscle relaxant, and central nervous system depressant properties, along with its therapeutic application in narcolepsy and alcohol dependence [37]. Guaiacol is notable among phenolic compounds for its expectorant, analgesic, and antiseptic qualities, as well as its anti-inflammatory and antioxidant capabilities [44]. Succinimide, found in both sections, is known for its anticonvulsant, sedative, and enzyme-inhibiting properties, particularly in neurological conditions [41]. Phenol, a thoroughly researched antiseptic and disinfectant, offers extra antibacterial, antifungal, and pain-relieving effects [46]. Additionally, 2-methoxy-4-vinylphenol and hydroquinone exhibit potent antioxidant, anti-inflammatory, and anticancer properties, with hydroquinone also recognized for its ability to depigment skin and inhibit melanin production [47,48], further underscoring the therapeutic potential of these constituents in dermatological, oncological, and inflammatory conditions, and supporting the pharmacological relevance of *A. nobilis* as a source of multifunctional bioactive compounds.

Compared to earlier pharmacological research on *A. nobilis* and its subspecies, our results provide significant new information, especially about the antimicrobial effectiveness of ethanol extracts from both aerial and root sections. Our findings highlighted that the aerial extract displayed more potent inhibitory effects on all examined microorganisms, with the lowest MIC values recorded against MRSA (0.5 mg/mL), *C. albicans* (0.75 mg/mL), and *C. neoformans* (0.85 mg/mL), suggesting noteworthy antibacterial and antifungal activity. Additionally, the aerial oil extract showed moderate antimicrobial potency, with MICs ranging from 0.9 mg/mL against *MRSA* to 3.2 mg/mL against *P. aeruginosa*. The lowest MICs of the oil extract were also observed against *MRSA* (0.9 mg/mL), *C. albicans* (1.2 mg/mL), and *C. neoformans* (1.4 mg/mL), supporting its potential as a lipophilic antimicrobial agent. This enhanced activity is likely associated with the higher abundance of key bioactive compounds identified by GC–MS. In particular, 4-hydroxybutanoic acid (7.76% in aerial vs. 2.34% in root), guaiacol (2.40% vs. 0.72%), and hydroquinone (1.74% vs. 1.35%), all of which are well-supported in the literature for their antimicrobial mechanisms. 4-hydroxybutanoic acid not only exerts central nervous system depressant effects and is used clinically in narcolepsy and alcohol dependence, but also modulates innate immunity by upregulating cathelicidin LL-37, activating GPR109A, and inducing MAP kinase/NF-κB pathways, thereby increasing resistance to microbial infections [38,39]. Guaiacol is recognized for its antiseptic, expectorant, and anti-inflammatory effects, and acts as a local anesthetic and antioxidant that contributes to membrane disruption in pathogens [44]. Hydroquinone, known for its melanin-inhibiting and antioxidant properties, also exhibits broad-spectrum antibacterial and antifungal activities and anti-inflammatory effects [49,67]. While these three compounds were highlighted due to both their abundance and mechanistic relevance, it is also likely that other phytochemicals—such as phenol, isosorbide, and 2-methoxy-4-vinylphenol—act synergistically to enhance the antimicrobial efficacy of the aerial extract. These results are consistent with earlier studies on essential oils and extracts of *A. nobilis* subspecies, including *sipylea* and *neilreichii*, which have shown strong activity against *C. albicans*, *E. faecalis*, and *P. vulgaris*, likely due to the presence of 1,8-cineole, fragranol, linalool, *α*-bisabolol, and fragranyl acetate [9,15,20,67]. Moreover, antibacterial effects against *E. coli*, *K. pneumoniae*, *P. aeruginosa*, and *S. aureus* were linked to high levels of *α*-thujone, artemisia ketone, and camphor [15], while phenolic-rich Soxhlet extracts of *A. nobilis* subsp. *neilreichii* demonstrated low MIC values against *S. aureus*, further supporting the antimicrobial potential of the genus [20].

Compared to other *Achillea* species, *Achillea nobilis* demonstrates notable but moderately selective antimicrobial activity, which can be attributed to its major compounds such as *α*-thujone, fragranol, and fragranyl acetate. However, when contrasted with *Achillea clavennae*, which exhibited the strongest antimicrobial activity among four tested species (*A. clavennae*, *A. holosericea*, *A. lingulata*, and *A. millefolium*), the extract of *A. clavennae* was more potent and yielded structurally diverse compounds including guaiane sesquiterpenes (rupicolin A and B, and their peroxide derivatives) and flavonoids like apigenin and centaureidin—compounds with well-documented antimicrobial and anti-inflammatory properties [68]. Furthermore, *A. atrata* showed superior bioactivity against both *Propionibacterium acnes* and *S. epidermidis*, with significant anti-MRSA potential. Its chemical profile was rich in polar phenolics such as syringetin-3-*O*-glucoside, mearnsetin-hexoside, and nevadensin, which were absent in *A. nobilis* [69]. Similarly, *A. wilhelmsii*, collected from Iran, exhibited a unique chemotype rich in oxygenated monoterpenes, particularly fragranol (33.2%) and fragranyl acetate (16.2%), and showed a broad antimicrobial spectrum including *C. albicans*, *S. aureus*, *Acinetobacter baumannii*, and *Shigella dysenteriae*. Its inhibitory halos (~9–10 mm) were comparable to those of rifampin, emphasizing its pharmacological potential [70]. While *A. nobilis* shares some chemical similarities with *A. wilhelmsii* (e.g., fragranol and fragranyl acetate), its antimicrobial range appears more limited in scope and intensity. Therefore, although *A. nobilis* exhibits valuable antimicrobial properties, other species such as *A. clavennae*, *A. atrata*, and *A. wilhelmsii* may offer broader or stronger bioactivity due to the presence of distinct sesquiterpenes and phenolic derivatives.

Leveraging the encouraging phytochemical and antimicrobial discoveries related to *A. nobilis*, our research group plans to continue experimental investigations aimed at enhancing its therapeutic capabilities through various specific strategies. As part of our ongoing and future work, bioassay-guided fractionation of ethanol extracts will be performed to isolate and identify the particular bioactive compounds responsible for the potent antimicrobial effects, specifically against MRSA, *C. albicans*, and *C. neoformans*. In vitro cytotoxicity studies and selectivity index evaluations will support these efforts to guarantee safety and therapeutic significance. Moreover, combinatory tests will be conducted to investigate potential synergistic interactions with traditional antimicrobial drugs. Transcriptomic and metabolomic analyses will be utilized to enhance the comprehension of the plant’s bioactivity by exploring the biosynthetic pathways that contribute to the production of pharmacologically important secondary metabolites. Seasonal and environmental factors affecting phytochemical content will be evaluated to determine ideal collection conditions for optimal effectiveness. Sophisticated computational methods like molecular docking and in silico ADMET modeling will be utilized to forecast the drug-likeness and action mechanisms of important compounds. In the end, the development of formulations using standardized extracts or purified components will be investigated for topical and oral therapeutic uses. These studies are part of our ongoing research agenda, and we are committed to validating *A. nobilis* as a scientifically reliable source of new antimicrobial compounds and promoting its inclusion in contemporary phytopharmaceutical advancement by integrating preclinical efficacy data, pharmacokinetic profiling, and scalable extraction techniques to facilitate its transition from traditional use to evidence-based medical applications.

## 4. Materials and Methods

### 4.1. Plant Collection and Identification

On 21 July 2024, plant samples of *A. nobilis* were methodically gathered from its native environment in the Aktobe region, situated in the western Kazakhstan, a semi-arid zone noted for varied steppe flora. Samples comprised both above-ground structures and root systems, collected specifically at the peak flowering period to guarantee optimal phytochemical yield, since this developmental stage is recognized for having the greatest concentration of secondary metabolites. After collection, all plant materials were thoroughly washed with distilled water to eliminate soil and debris, and then air-dried in the shade at room temperature (22–25 °C) for 10–14 days to protect thermolabile compounds. The dried substance was then ground with a mechanical grinder to achieve a uniform fine powder, which was kept in sealed containers in dark, dry conditions until extraction. This preparatory process was performed following standard pharmacognostic protocols to preserve chemical integrity and guarantee the reproducibility of subsequent phytochemical analyses.

### 4.2. Chemicals and Reagents

All chemicals and reagents used in this research were of analytical quality to guarantee the consistency and repeatability of experimental findings. Ethanol (96%, *v*/*v*) served as the main solvent for extracting phytoconstituents, owing to its effectiveness in dissolving various polar and moderately non-polar substances. For qualitative phytochemical analysis, reagents were prepared following standard procedures: Dragendorff’s reagent (Sigma-Aldrich, St. Louis, MO, USA) was used to identify alkaloids, gelatin solution for tannins, ferric chloride (Sigma-Aldrich, St. Louis, MO, USA) for flavonoids, Liebermann–Burchard reagent (Sigma-Aldrich, St. Louis, MO, USA) for triterpenoids, and the Keller–Killiani test (using glacial acetic acid and ferric chloride from Merck, Darmstadt, Germany) for cardiac glycoside detection. Reagents were freshly made and kept in amber containers under regulated laboratory conditions to avoid photodegradation. For antimicrobial tests, culture media such as Mueller–Hinton Broth (MHB), Mueller–Hinton Agar (MHA), Sabouraud Dextrose Agar (SDA), and RPMI-1640 medium were obtained from HiMedia Laboratories (Mumbai, India), guaranteeing standardization in microbial susceptibility assessments. Dimethyl sulfoxide (DMSO, ≥99.9%, Sigma-Aldrich, St. Louis, MO, USA) was utilized as the solvent to create extract stock solutions for antimicrobial assessment, due to its capacity to dissolve a wide range of organic substances without affecting microbial growth. Helium gas of high purity (99.999%, Linde Gas, Munich, Germany) served as the carrier gas in the gas chromatography–mass spectrometry (GC–MS) analysis, guaranteeing superior resolution and sensitivity. The identification of compounds in GC–MS was aided by comparing spectra with the mass spectral libraries of the National Institute of Standards and Technology (NIST’02, Gaithersburg, MD, USA) and Wiley 7. All solutions and media were prepared with double-distilled water and stored under suitable laboratory conditions (4–8 °C for reagents and 20–25 °C for solvents and media) to maintain their chemical stability and analytical integrity.

### 4.3. Vortex-Assisted Extraction Method (VAM)

The VAM was utilized to obtain bioactive compounds from botanical sources. In this process, 70 g of finely ground raw materials was utilized, with the solvent comprising 340 mL of 96% ethyl alcohol. The extraction procedure was conducted at room temperature for a period of 110 min. Throughout this period, the vortex mixer enabled swift mixing of the solvent and plant material, aiding in efficient mass transfer and improving the solubility of the desired compounds. The rapid vortex motion guaranteed that the extraction solvent consistently engaged with the raw materials, enhancing the extraction efficiency. Following the extraction phase, the resulting solution underwent filtration with either standard filter paper or a vacuum filtration system to separate the intended extract. The filtrate was gathered, and the leftover solid material was thrown away. Following the filtration of the extract acquired via the VAM procedure, the filtrate underwent pre-concentration with a rotary evaporator at a temperature that did not surpass 40 °C under reduced pressure to eliminate most of the ethanol solvent. This temperature was carefully selected based on prior studies demonstrating that key phytochemical constituents, including flavonoids, triterpenoids, and glycosides, remain stable below 40°C during evaporation, minimizing degradation and ensuring the integrity of thermolabile compounds [71,72]. The concentrated extract was frozen at −80 °C for 12 h to guarantee full solidification. The frozen extract was subjected to freeze-drying (Labconco FreeZone 2.5, Kansas City, MO, USA), enabling solvent sublimation under a vacuum of around 0.05 Mbar at a chamber temperature of −50 °C. Remaining moisture was eliminated during the secondary drying stage at ~20 °C. The obtained powdered extract was kept in airtight containers and safeguarded from moisture for later phytochemical and bioactivity evaluations.

### 4.4. Oil-Based Maceration Extraction

To obtain the oil extract of the aerial parts of *A. nobilis*, a cold maceration technique was employed using sunflower oil as the solvent, in accordance with established phytochemical extraction protocols [73,74]. Briefly, 50 g of air-dried and finely powdered aerial plant material was immersed in 500 mL of refined sunflower oil in a sealed glass container. The mixture was kept at room temperature (25 ± 2 °C) for 14 days, protected from direct sunlight, and stirred gently once daily to facilitate diffusion of lipophilic phytochemicals into the solvent. Following the extraction period, the macerate was filtered through muslin cloth and subsequently through Whatman No. 1 filter paper to remove solid residues. The resulting oil extract was stored in amber glass bottles at 4 °C until further phytochemical analysis. This extraction method was specifically chosen to isolate non-polar, lipid-soluble bioactive constituents such as fatty acids, terpenes, sterols, and hydrocarbons, which are not efficiently recovered through ethanol-based extractions. In addition to its chemical relevance, the oil extract also aligns with traditional ethnopharmacological uses of *A. nobilis* for dermal applications, serving as a practical basis for topical phytotherapeutic formulations.

### 4.5. Preliminary Qualitative Analysis

The initial phytochemical analysis of *A. nobilis* aerial and root ethanol extracts was conducted utilizing standardized colorimetric and precipitation assays to qualitatively identify prominent secondary metabolites, as delineated by Barros et al. [75], with minor adaptations. The presence of alcohols was indicated by the emergence of a pronounced blue or green hue upon reaction with ferric chloride, while the presence of aldehydes resulted in a pink-to-magenta coloration upon treatment with Schiff’s reagent. The detection of amines was achieved through the use of ninhydrin reagent, which elicits a purple coloration in the presence of either primary or secondary amines. The confirmation of amides was accomplished by heating the extract with sodium hydroxide, with the liberation of gas or an ammonia-like odor serving as an indicator of a positive outcome. Flavonoids were evaluated utilizing ferric chloride, followed by the addition of hydrochloric acid, where the fleeting yellow coloration that subsequently dissipated validated their presence. The assessment of tannins was conducted using gelatin reagent, where the formation of dirty brownish-green precipitates was indicative of a positive test result. Alkaloids were identified through the development of a reddish-orange precipitate following the addition of Dragendorff’s reagent. The confirmation of triterpenoids was achieved via the Liebermann–Burchard reaction, characterized by the appearance of a brown ring, while glycosides were detected through the Keller–Killiani test, which resulted in a reddish-brown layer. All assays were executed in triplicate, and the results were interpreted visually based on distinct colorimetric changes or the formation of precipitates.

### 4.6. GC–MS Profiling

GC–MS analyses were performed separately for the ethanol extracts from the aerial and root parts, as well as for the essential oil extracted from the aerial part of *A. nobilis*. Each sample type was analyzed using optimized conditions tailored to its physicochemical properties to ensure accurate compound separation and identification.

The ethanol extracts of both aerial and root parts were analyzed using a Shimadzu GC–MS system (Shimadzu Corporation, Kyoto, Japan) equipped with a DB-35 MS ultra-inert capillary column (30 m × 0.25 mm, film thickness: 0.25 μm). The oven temperature program was set to begin at 40 °C (held for 3 min), followed by a linear increase to 280 °C at a rate of 5 °C/min, and then held isothermally for an additional 15 min. The injection port was maintained at 280 °C, with helium (99.999%) as the carrier gas at a constant flow rate of 1.4 mL/min. Extracts were diluted to 1% (*v*/*v*) and injected in split mode (15:1), with a sample volume of 1 μL. The mass spectrometer operated in electron impact ionization mode (70 eV), with the ion source and interface temperatures set at 220 °C and 280 °C, respectively. Full scan mode was used over the m/z range of 34–750. Compound identification was based on spectral comparison with reference databases (NIST’02 and Wiley 7) using Agilent MSD ChemStation (version 1701EA, Santa Clara, CA, USA).

The aerial oil extract was analyzed using a system configured with an Rtx-100DHA capillary column (30 m × 0.25 mm, film thickness: 0.5 μm, Restek, Bellefonte, PA, USA). The temperature program was initiated at 60 °C and increased to 300 °C at 8 °C/min. The injector temperature was set at 280 °C, while the ion source and quadrupole temperatures were maintained at 230 °C and 150 °C, respectively. Helium served as the carrier gas at a column pressure of 2 psi. A 0.2 μL sample was injected in split mode, and mass spectra were recorded in full scan mode. The total runtime for analysis was 32 min. Compound identification was performed using the NIST 08 spectral library, and data processing was carried out using GS-MSD Data Analysis software (version G1701EA E.02.02.1431, Agilent Technologies, Santa Clara, CA, USA). The relative abundance of compounds was estimated using a semi-quantitative approach based on peak area percentages.

### 4.7. Antimicrobial Activity

The antimicrobial properties of ethanol extracts derived from the aerial and root components of *A. nobilis* were assessed against a range of eight clinically significant microbial strains, including both bacterial and fungal pathogens. The organisms tested comprised methicillin-resistant *S. aureus* (MRSA, ATCC 43300), vancomycin-resistant *E. faecalis* (VRE, ATCC 51299), *E. coli* (ATCC 25922), *P. aeruginosa* (ATCC 27853), *K. pneumoniae* (ATCC 700603), *C. albicans* (ATCC 90028), *A. fumigatus* (ATCC 1022), and *C. neoformans* (ATCC 208821). The broth microdilution method was employed to ascertain the MICs of the extracts, following the Clinical and Laboratory Standards Institute (CLSI) guidelines released in 2018. Bacterial strains were preserved on MHA, whereas fungal isolates were cultivated on Sabouraud Dextrose Agar (SDA), with all cultures incubated at 37 °C for 24 to 48 h to promote optimal growth. Microbial inocula were adjusted to a turbidity matching 0.5 McFarland standard, which is roughly 1 × 10^8^ colony-forming units per milliliter (CFU/mL), and then diluted to obtain a final working concentration of 1 × 10^5^ CFU/mL in every well. Two-fold serial dilutions of the plant extracts, from 90 to 5000 µg/mL, were made in sterile 96-well U-bottom microplates using MHB for bacterial tests and RPMI-1640 medium for fungal tests [76,77]. Prior to MIC testing, the solubility of the extracts at the highest concentration (5 mg/mL) in the respective media was visually confirmed to ensure complete dissolution and avoid precipitation during the assay. The experimental design featured negative controls (extract in medium absent of microorganisms) and positive controls (medium containing microbial inoculum but lacking extract). After incubation at 35 °C for 24 to 48 h, MIC values were noted as the minimum extract concentrations that entirely prevented observable microbial growth under typical laboratory lighting conditions. This method facilitated a comparative evaluation of the inhibitory capacity of aerial and root-derived extracts against multidrug-resistant and opportunistic pathogens, while the use of RPMI-1640 for fungal MIC determination was based on CLSI M27-A3 [78] guidelines, which recommend this medium for standardizing antifungal susceptibility testing of yeasts and molds [77].

### 4.8. Statistical Analysis

All data are expressed as mean values accompanied by their corresponding standard deviations (mean ± SD). Statistical significance of the results was determined using Student’s *t*-test and one-way analysis of variance (ANOVA) to assess differences between experimental groups.

## 5. Conclusions

This research provides a comprehensive phytochemical and antimicrobial assessment of *A. nobilis*, incorporating for the first time a comparative analysis of ethanol extracts from both aerial and root parts alongside a macerated aerial oil extract. Through qualitative screening and GC–MS profiling, a chemically diverse array of bioactive secondary metabolites was identified, including phenolics, alcohols, organic acids, lactones, and lipophilic compounds such as sterols, terpenoids, and unsaturated fatty acids. Many of these constituents are known for their antimicrobial, antioxidant, anti-inflammatory, and neuroprotective effects.

Significantly, the aerial ethanol extract demonstrated the most potent antimicrobial activity, particularly against critical pathogens such as *MRSA*, *C. albicans*, and *C. neoformans*, supporting its promising therapeutic relevance. The root ethanol extract, while exhibiting comparatively lower potency, still revealed noteworthy antimicrobial effects, consistent with the traditional holistic use of the entire plant. Importantly, the inclusion of the aerial oil extract revealed a distinct and pharmacologically meaningful profile rich in lipid-soluble compounds, including *α*-tocopherol, linoleic acid, camphor, and squalene—substances known for their strong antioxidant, anti-inflammatory, and antimicrobial activities. While its antimicrobial activity was moderate compared to the ethanol extract, the oil extract’s chemical profile aligns with its traditional use in topical and dermal applications and suggests specific potential in the development of lipid-based formulations.

Taken together, this study highlights the complementary therapeutic value of both polar and non-polar extracts from *A. nobilis* and expands the understanding of its bioactive spectrum. The findings are consistent with prior pharmacological reports on *A. nobilis* and its subspecies, further supporting its ethnomedicinal importance and broad biological potential, including anticonvulsant, antioxidant, antispasmodic, and anti-inflammatory effects. These results not only validate the traditional uses of *A. nobilis* but also establish a strong foundation for future research focused on bioassay-guided fractionation, elucidation of molecular mechanisms, and the development of both systemic and topical phytotherapeutic products. In conclusion, *A. nobilis* remains an underexplored medicinal plant with considerable pharmacological promise, meriting continued investigation for its integration into evidence-based herbal medicine and its role in the discovery of novel natural antimicrobial agents.

## Figures and Tables

**Table 1:** Phytochemical screening of aerial and root extracts of *A. nobilis*.

Phytochemical Groups	Reagent	Observation	Aerial Ethanol Extract	Aerial Oil Extract	Root Ethanol Extract
Alcohols	Ferric chloride	Intense blue or green coloration	+	+	+
Aldehydes	Schiff’s reagent	Pink to magenta color formation	+	+	+
Amines	Ninhydrin reagent	Purple coloration	+	−	+
Amides	Sodium hydroxide + heat	Ammonia-like smell or evolution of gas	+	−	+
Flavonoids	Ferric chloride	Yellowish appearance clears after acid (HCl) addition	+	−	+
Tannins	Gelatin	Dirty (brownish) green precipitates	−	−	+
Alkaloids	Dragendorff’s	Reddish-orange precipitate	+	−	+
Triterpenoids	Liebermann–Burchard	Brown ring	+	+	+
Glycosides	Keller–Killiani	Reddish-brown layer	+	−	+

“+” indicates the confirmed presence, while “–“ denotes the absence of the respective phytochemical group in the aerial and root extracts of *A. nobilis* based on preliminary phytochemical screening.

**Table 2:** Characterization of aerial ethanol extract constituents of *A. nobilis* through the GC–MS technique.

No.	Name	Molecular Formula	Molecular Mass, g/mol	Retention Indices (RI)	Retention Time (min)	PubChem Compound CID	Similarities	Area, %
1	Propanoic acid	C_3_H_6_O_2_	74.08	878	10.58	1032	93	0.92
2	2,3-Butanediol	C_4_H_10_O_2_	90.12	1055	11.40	262	96	6.08
3	*R*-(–)-1,2-propanediol	C_3_H_8_O_2_	76.09	946	11.62	259994	91	0.89
4	Gamma-Butyrolactone	C_4_H_6_O_2_	86.09	960	12.32	7302	96	0.95
5	Butanoic acid	C_4_H_8_O_2_	88.11	950	12.40	264	95	1.89
6	4-Hydroxybutanoic acid	C_4_H_8_O_3_	104.1	1026	12.99	10413	96	7.76
7	(L)-*α*-Terpineol	C_10_H_18_O	154.25	1195	14.22	443162	92	1.53
8	N-Nitrosohexamethyleneimine	C_6_H_12_N_2_O	128.17	1120	14.35	13613	90	0.19
9	1,2-Cyclopentanedione	C_5_H_6_O_2_	98.1	985	15.28	566657	89	0.54
10	Methyl N-hydroxybenzenecarboximidate	C_8_H_9_NO_2_	151.16	1210	15.58	9602988	98	2.69
11	Guaiacol	C_7_H_8_O_2_	124.14	1105	17.01	460	85	2.40
12	Ethanol, 2,2’-oxybis-	C_4_H_10_O_3_	106.12	1035	19.14	161927	87	1.82
13	Phenol	C_6_H_6_O	94.11	1080	19.77	996	90	2.01
14	Phenol, 4-ethyl-2-methoxy-	C_9_H_12_O_2_	152.19	1255	20.35	62465	87	1.08
15	2-Pyrrolidinone	C_4_H_7_NO	85.1	1010	20.65	12025	97	3.22
16	1,3-Propanediol	C_3_H_8_O_2_	76.09	920	22.57	10442	95	2.20
17	Eugenol	C_10_H_12_O_2_	164.2	1356	22.75	3314	87	1.49
18	2-Methoxy-4-vinylphenol	C_9_H_10_O_2_	150.17	1320	23.43	332	85	3.26
19	Dianhydromannitol	C_6_H_10_O_4_	146.14	1350	23.66	23619611	95	1.07
20	Phenol, 2,6-dimethoxy-	C_8_H_10_O_3_	154.16	1280	24.66	7041	85	2.85
21	Glycerin	C_3_H_8_O_3_	92.09	1150	25.04	753	86	15.97
22	Triethylene glycol	C_6_H_14_O_4_	150.17	1175	25.33	8172	91	2.80
23	1,4:3,6-Dianhydro- *α*-*d*-glucopyranose	C_6_H_8_O_4_	144.13	1385	26.35	22213879	87	1.76
24	Benzofuran, 2,3-dihydro-	C_8_H_8_O	120.15	1150	26.75	10329	88	0.60
25	5-tert-Butylpyrogallol	C_10_H_14_O_3_	182.22	1400	27.08	597592	83	0.60
26	Succinimide	C_4_H_5_NO_2_	99.09	990	27.62	11439	94	0.48
27	Isosorbide	C_6_H_10_O_4_	146.14	1310	29.53	12597	90	7.91
28	3’,5’-Dimethoxyacetophenone	C_10_H_12_O_3_	180.2	1450	29.73	95997	86	2.54
29	Tetraethylene glycol	C_8_H_18_O_5_	194.23	1225	31.20	8200	96	3.22
30	3-Methyl-4-phenyl-1H-pyrrole	C_11_H_11_N	157.21	1420	31.98	15164561	85	0.61
31	Hydroquinone	C_6_H_6_O_2_	110.11	1155	35.97	785	89	1.74
32	3-Isobutylhexahydropyrrolo [1,2-a]pyrazine-1,4-dione	C_11_H_18_N_2_O_2_	210.27	1325	36.69	102892	87	1.27
33	Hexaethylene glycol	C_12_H_26_O_7_	282.33	1275	37.18	17472	88	3.56
34	Octadecanoic acid	C_18_H_36_O_2_	284.48	1980	37.75	5281	93	1.42
35	Pyrrolo[1,2-a]pyrazine-1,4-dione, hexahydro-3-(2-methylpropyl)-	C_10_H_16_N_2_O_2_	196.25	1350	38.65	98951	84	2.19
36	*β*-D-Glucopyranose, 1,6-anhydro-	C_6_H_10_O_5_	162.14	1400	39.66	11947765	83	1.39
37	Pyrrolo[1,2-a]pyrazine-1,4-dione, hexahydro-	C_7_H_10_N_2_O_2_	154.17	1295	41.55	193540	84	1.07
38	4-Methyleneproline	C_6_H_9_NO_2_	127.14	1230	45.85	558375	86	5.63

**Table 3:** GC–MS identification of phytochemicals in the root ethanol extract of *A. nobilis.*

No.	Name	Molecular Formula	Molecular Mass, g/mol	Retention Indices (RI)	Retention Time (min)	PubChem Compound CID	Similarities	Area, %
1	Propanoic acid	C_3_H_6_O_2_	74.08	880	10.52	1032	85	0.96
2	4-Cyclopentene-1,3-dione	C_5_H_4_O_2_	96.08	900	11.27	70258	80	0.81
3	4-Hydroxybutanoic acid	C_4_H_8_O_3_	104.10	920	12.22	10413	82	2.34
4	2-Propenoic acid	C_3_H_4_O_2_	72.06	940	12.48	6581	85	1.16
5	2-Furanmethanol	C_5_H_6_O_2_	98.10	960	12.92	7361	86	1.14
6	2(5H)-Furanone, 3-methyl-	C_5_H_6_O_2_	98.10	980	14.16	30945	82	0.39
7	2,4-Dimethyl-2-oxazoline-4-methanol	C_6_H_11_NO_2_	129.16	1000	14.26	98073	80	3.01
8	2(5H)-Furanone	C_4_H_4_O_2_	84.07	1020	14.69	10341	82	0.97
9	1,2-Cyclopentanedione	C_5_H_6_O_2_	98.1	1040	15.16	566657	85	2.55
10	1,2-Cyclopentanedione, 3-methyl-	C_6_H_8_O_2_	112.13	1060	16.54	61209	94	0.69
11	Guaiacol	C_7_H_8_O_2_	124.14	1080	16.98	460	88	0.72
12	Benzaldehyde, 3-hydroxy-, oxime	C_7_H_7_NO_2_	137.14	1100	17.49	9603073	86	6.65
13	2-Cyclopenten-1-one, 3-ethyl-2-hydroxy-	C_7_H_10_O_2_	126.15	1120	17.84	62752	82	0.54
14	Ethanone, 1-(1H-pyrrol-2-yl)-	C_6_H_7_NO	109.13	1140	19.14	14079	80	0.45
15	Phenol	C_6_H_6_O	94.11	1160	19.75	996	82	0.61
16	2-Pyrrolidinone	C_4_H_7_NO	85.10	1180	20.68	12025	85	0.65
17	2(3H)-Furanone, 5-heptyldihydro-	C_11_H_18_O_2_	182.26	1200	20.86	7714	94	0.70
18	Cyclopropyl carbinol	C_4_H_8_O	72.11	1220	21.04	75644	88	1.06
19	1,3-Dioxol-2-one,4,5-dimethyl-	C_5_H_6_O_3_	114.10	1240	21.79	142210	90	1.39
20	*α*-Hydroxy-gamma-butyrolactone	C_4_H_6_O_3_	102.09	1260	22.53	19444	85	1.26
21	Allyl acetate	C_5_H_8_O_2_	100.12	1280	22.61	11584	80	0.85
22	2-Methoxy-4-vinylphenol	C_9_H_10_O_2_	150.17	1300	23.38	332	82	1.10
23	2,3-Dimethylhydroquinone	C_8_H_10_O_2_	138.17	1320	23.95	69100	85	0.41
24	4H-Pyran-4-one, 2,3-dihydro-3,5-dihydroxy-6-methyl-	C_6_H_6_O_4_	142.11	1340	24.12	119838	94	9.39
25	1,2,3-Propanetriol, 1-acetate	C_5_H_10_O_4_	134.13	1360	24.58	33510	88	0.42
26	Phenol, 2,6-dimethoxy-	C_8_H_10_O_3_	154.16	1380	24.65	7041	80	1.57
27	Glycerin	C_3_H_8_O_3_	92.09	1400	25.21	753	82	26.73
28	Triethylene glycol	C_6_H_14_O_4_	150.17	1420	25.36	8172	85	1.05
29	1,4:3,6-Dianhydro-*α*-*d*-glucopyranose	C_6_H_8_O_4_	144.13	1440	26.35	39923607	94	0.40
30	Benzofuran, 2,3-dihydro-	C_8_H_8_O	120.15	1460	26.72	10329	85	0.41
31	Acetaminophen	C_8_H_9_NO_2_	151.16	1480	26.91	1983	88	0.09
32	Benzoic acid, 3-pyridyl ester	C_12_H_9_NO_2_	199.20	1500	27.10	569697	87	1.88
33	Succinimide	C_4_H_5_NO_2_	99.09	1520	27.59	11439	76	0.34
34	(S)-(+)-2’,3’-Dideoxyribonolactone	C_4_H_6_O_3_	102.09	1540	27.76	32780	89	0.96
35	2-Aminopyrimidine-1-oxide	C4H5N3O	111.10	1560	28.04	139694	80	0.53
36	2,5-Dimethyl-4-phenylpyridine	C_13_H_13_N	183.25	1580	28.64	603086	82	0.24
37	3-Pyridinol, 6-methyl-	C_6_H_7_NO	109.13	1600	28.81	14275	85	0.32
38	Butyl 9-decenoate	C_14_H_26_O_2_	226.36	1620	29.17	17825102	94	1.58
39	Isosorbide	C_6_H_10_O_4_	146.14	1640	29.54	12597	88	0.31
40	3’,5’-Dimethoxyacetophenone	C_10_H_12_O_3_	180.20	1660	29.68	95997	80	9.97
41	DL-Proline, 5-oxo-, methyl ester	C_6_H_9_NO_3_	143.14	1680	29.99	500249	82	0.12
42	Tetraethylene glycol	C_8_H_18_O_5_	194.23	1700	31.20	8200	85	1.95
43	3-Methyl-4-phenyl-1H-pyrrole	C_11_H_11_N	157.21	1720	31.97	15164561	86	0.48
44	3-(1H-Pyrrol-3-yl)propionic acid, methyl ester	C_8_H_11_NO_2_	153.18	1740	32.09	556813	92	0.46
45	2,6-Dimethylphenyl isocyanate	C_9_H_9_NO	147.17	1760	32.19	98787	86	0.46
46	Ethyl N-(o-anisyl)formimidate	C_10_H_13_NO_2_	179.22	1780	32.43	601627	90	0.38
47	2-Naphthalenamine	C_10_H_9_N	143.18	1800	33.40	7057	83	0.19
48	Benzaldehyde, 4-hydroxy-3,5-dimethoxy-	C_9_H_10_O_4_	182.17	1820	35.06	8655	98	0.38
49	Hydroquinone	C_6_H_6_O_2_	110.11	1840	35.95	785	93	1.35
50	Pentaethylene glycol	C_10_H_22_O_6_	238.28	1860	37.18	62551	89	0.50
51	Uric acid	C_5_H_4_N_4_O_3_	168.11	1880	37.83	1175	93	0.70
52	Pyrrolo[1,2-a]pyrazine-1,4-dione, hexahydro-3-(2-methylpropyl)-	C_11_H_18_N_2_O_2_	210.27	1900	37.99	98951	86	1.55
53	4-((1E)-3-Hydroxy-1-propenyl)-2-methoxyphenol	C_10_H_12_O_3_	180.20	1920	39.02	1549095	88	0.59
54	*β*-D-Glucopyranose, 1,6-anhydro-	C_6_H_10_O_5_	162.14	1940	39.66	11947765	89	0.33
55	Pyrrolo[1,2-a]pyrazine-1,4-dione, hexahydro-	C_7_H_10_N_2_O_2_	154.17	1960	41.53	193540	93	0.78
56	Hexaethylene glycol	C_12_H_26_O_7_	282.33	1980	42.43	17472	86	0.45
57	Uracil	C_4_H_4_N_2_O_2_	112.09	2000	45.19	1174	88	0.56
58	4-Methyleneproline	C_6_H_9_NO_2_	127.14	2020	45.88	558375	98	2.25

**Table 4:** Characterization of aerial oil extract constituents of *A. nobilis* through the GC–MS technique.

No.	Name	Molecular Formula	Molecular Mass, g/mol	Retention Indices (RI)	Retention Time (min)	PubChem Compound CID	Similarities	Area, %
1	Camphor	C_10_H_16_O	152.23	1173	10.07	2537	93	1.41
2	2,4-Decadienal	C_10_H_16_O	152.23	1280	12.61	5283349	96	0.46
3	2-Furanacetaldehyde, *α*-propyl-	C_10_H_12_O_2_	152.19	1076	12.99	557292	91	0.69
4	3-(Hydroxymethylene)indolin-2-one	C_9_H_7_NO_2_	161.16	1490	16.21	595118	96	0.66
5	Hedycaryol	C_15_H_26_O	222.37	1534	18.67	6432240	95	0.70
6	6-Methyl-2,4(1H,3H)-pteridinedione	C_7_H_6_N_4_O_2_	178.15	1590	20.27	601068	96	0.57
7	Tridecanoic acid, methyl ester	C_4_H_28_O_2_	228.37	1608	22.03	15608	92	0.92
8	Hexadecanoic acid, ethyl ester	C_18_H_36_O_2_	284.50	1994	22.87	12366	86	1.15
9	11,14-Octadecadienoic acid, methyl ester	C_19_H_34_O_2_	294.50	2089	24.03	5365677	90	0.68
10	4-Methyl-3-pentenal	C_6_H_10_O	98.14	942	24.13	21457	92	0.60
11	6-Tetradecyne	C_14_H_26_	194.36	1395	24.43	138027	87	14.99
12	Linoleic acid	C_18_H_32_O_2_	280.40	2095	24.52	5280450	85	8.08
13	Linoleic acid ethyl ester	C_20_H_36_O_2_	308.50	2145	24.80	5282184	93	1.75
14	Oleic acid ethyl ester	C_20_H_38_O_2_	310.50	2175	24.89	5363269	88	2.28
15	2,2,2-Trifluoro-N-(hydroxymethyl)acetamide	C_3_H_4_F_3_NO_2_	143.06	2197	25.66	3084931	95	3.35
16	Palmitoyl chloride	C_16_H_34_ClO	274.90	2256	26.19	8206	91	2.50
17	*α* -Tocopherol	C_29_H_50_O_2_	430.70	3100	27.26	14985	86	8.36
18	*E*,*Z*-1,3,12-Nonadecatriene	C_19_H_34_	262.50	1916	27.44	5365680	88	22.25
19	Oleic acid, 3-hydroxypropyl ester	C_21_H_40_O_3_	340.50	2076	27.51	5352775	90	6.92
20	2-Dodecylcyclobutanone	C_16_H_30_O	238.41	1600	27.77	161875	90	1.24
21	Isopropyl linoleate	C_21_H_38_O_2_	322.50	2150	27.96	5352860	81	14.64
22	*cis*-13,16-Docasadienoic acid	C_22_H_40_O_2_	336.60	2566	28.28	5312554	91	1.42
23	1-(Trimethylsilyl)-1-propyne	C_6_H_12_Si	112.240	2509	30.71	80363	85	0.49
24	Chondrillasterol	C_29_H_48_O	412.70	3380	30.86	5283663	89	1.54
25	Squalene	C_30_H_50_	410.70	2814	31.65	638072	90	2.35

**Table 5:** Identified phytochemicals in aerial and root extracts of *A. nobilis*.

No.	Chemical Class	Subclass	Name	Known Pharmacological Activities
1.	Alcohol	Diol	2,3-Butanediol ^a^	Antitumor activity, immunomodulatory effects, cryoprotective agent, solvent and drug carrier, anti-inflammatory properties, neuroprotective effects, probiotic metabolite potential, inhibitory effect on certain pathogens [26,27]
2.		Diol	*R*-(–)-1,2-propanediol ^a^	–
3.		Diol	1,3-Propanediol ^a^	Solvent and drug delivery agent, moisturizing and humectant properties, stabilizing agent [28]
4.		Monoterpene alcohol	(L)-*α*-Terpineol ^a^	–
5.		Triol	Glycerin ^a,r^	Humectant and lubricant [29]
6.		Polyether diol	Triethylene glycol ^a,r^	Antimicrobial activity, antiviral activity, disinfectant properties, low toxicity profile, plasticizer and solvent in pharmaceuticals, air sanitizing agent, humectant in topical formulations [30]
7.		Polyether diol	Tetraethylene glycol ^a,r^	–
8.		Polyether diol	Hexaethylene glycol ^a,r^	–
9.		Furan derivative	2-Furanmethanol ^r^	Antimicrobial, antifungal, and anticancer [31]
10.		Cyclopropyl alcohol	Cyclopropyl carbinol ^r^	–
11.		Polyether	Pentaethylene glycol ^r^	–
12.	Aldehyde	Oxime	Benzaldehyde, 3-hydroxy-, oxime ^r^	–
13.		*α*,*β*-Unsaturated aliphatic aldehyde	2,4-Decadienal ^b^	–
14.		*α*,*β*-Unsaturated aldehyde	4-Methyl-3-pentenal ^b^	–
15.		Phenolic aldehyde	Benzaldehyde, 4-hydroxy-3,5-dimethoxy- ^r^	–
16.		Furan derivative	2-Furanacetaldehyde, *α*-propyl- ^b^	–
17.	Aromatic compound	Benzofuran derivative	Benzofuran, 2,3-dihydro- ^a,r^	–
18.	Amino acid	Non-proteinogenic amino acid	4-Methyleneproline ^b^	–
19.	Amino acid derivative	Lactam ester	DL-Proline, 5-oxo-, methyl ester ^r^	–
20.	Amine	Aromatic amine	2-Naphthalenamine ^r^	–
21.	Amide	Aniline derivative	Acetaminophen ^r^	–
22.		Trifluoroacetamide derivative	2,2,2-Trifluoro-N-(hydroxymethyl)acetamide ^b^	–
23.	Carboxylic acid	Short-chain fatty acid	Propanoic acid ^a,r^	Antimicrobial activity, anti-inflammatory properties, anticancer potential, lipid metabolism regulation, gut microbiota modulation, histone deacetylase (HDAC) inhibition, immune response modulation [32]
24.		Short-chain fatty acid	Butanoic acid ^a^	HDAC inhibition [33]
25.		Alpha, beta-unsaturated acid	2-Propenoic acid ^r^	–
26.		Saturated fatty acid	Octadecanoic acid ^a^	Antimicrobial activity, anti-inflammatory properties, antioxidant activity, anticancer potential, emollient and skin-conditioning agent, cholesterol-lowering effects, immune-modulating activity [34]
27.		Polyunsaturated fatty acid	Linoleic acid ^b^	Anti-proliferative, anti-invasive, pro-apoptotic, cell cycle arrest (G1 phase), ROS-inducing, mitochondrial membrane potential disruption, anti-inflammatory, antioxidant, epithelial-mesenchymal transition (EMT) inhibition, angiogenesis inhibition, immune modulation, mitochondrial biogenesis stimulation, PGC-1*α*/NRF1/TFAM pathway activation [34,35]
28.		Polyunsaturated fatty acid	*cis*-13,16-Docasadienoic acid ^b^	–
29.		*γ*-hydroxy acid	4-hydroxybutanoic acid ^a,r^	CNS depressant activity, sedative effects, anesthetic properties, muscle relaxant, euphoric effects [36], treatment of narcolepsy, treatment of alcohol dependence, potential neuroprotective effects [37], upregulation of expression of the *Cramp* gene (encoding cathelicidin LL-37) in murine bone marrow-derived macrophages [38], promotion of endogenous antimicrobial peptide expression in macrophages [39]
30.	Carboxylic acid ester	Pyrrole derivative	3-(1H-Pyrrol-3-yl)propionic acid, methyl ester ^r^	–
31.		Saturated methyl ester	Tridecanoic acid, methyl ester ^b^	–
32.		Saturated ethyl ester	Hexadecanoic acid, ethyl ester ^b^	–
33.		Polyunsaturated methyl ester	11,14-Octadecadienoic acid, methyl ester ^b^	–
34.		Polyunsaturated ethyl ester	Linoleic acid ethyl ester ^b^	–
35.		Monounsaturated ethyl ester	Oleic acid ethyl ester ^b^	–
36.		Polyunsaturated ester	Isopropyl linoleate ^b^	–
37.		Monounsaturated ester	Oleic acid, 3-hydroxypropyl ester ^b^	–
38.	Carbohydrate	Dianhydrosugar alcohol	Dianhydromannitol ^a^	Diuretic activity, osmotic laxative effect, low toxicity, potential use as a pharmaceutical excipient, stabilizing agent, osmoprotective properties [40]
39.		Sugar derivative	1,4:3,6-Dianhydro-*α*-d-glucopyranose ^a,r^	–
40.		Sugar alcohol derivative	Isosorbide ^a,r^	–
41.		Monosaccharide derivative	*β*-D-Glucopyranose, 1,6-anhydro- ^a,r^	–
42.	Carbonate	Dioxolone	1,3-Dioxol-2-one,4,5-dimethyl- ^r^	–
43.	Ether	Diol ether	Ethanol, 2,2’-oxybis- ^a^	–
44.		Allyl ester	Allyl acetate ^r^	–
45.		Acylglycerol	1,2,3-Propanetriol, 1-acetate ^r^	–
46.		Pyridine ester	Benzoic acid, 3-pyridyl ester ^r^	–
47.		Fatty acid ester	Butyl 9-decenoate ^r^	–
48.	Heterocycle	Lactam	2-Pyrrolidinone ^a,r^	–
49.		Pyrrole derivative	3-Methyl-4-phenyl-1H-pyrrole ^a,r^	–
50.		Lactam	3- Isobutylhexahydropyrrolo[1,2-a]pyrazine-1,4-dione ^a^	–
51.		Lactam	Pyrrolo[1,2-a]pyrazine-1,4-dione, hexahydro-3-(2-methylpropyl)- ^a,r^	–
52.		Lactam	Pyrrolo[1,2-a]pyrazine-1,4-dione, hexahydro- ^a,r^	–
53.		Oxazoline derivative	2,4-Dimethyl-2-oxazoline-4-methanol ^r^	–
54.		Pyrimidine derivative	2-Aminopyrimidine-1-oxide ^r^	–
55.		Pyridine derivative	2,5-Dimethyl-4-phenylpyridine ^r^	–
56.		Indole derivative	3-(Hydroxymethylene)indolin-2-one ^b^	–
57.		Pteridine derivative	6-Methyl-2,4(1H,3H)-pteridinedione ^b^	–
58.	Imidate	Aromatic imidate	Methyl N-hydroxybenzenecarboximidate ^a^	–
59.		Aromatic imidate	Ethyl N-(o-anisyl)formimidate ^r^	–
60.	Imide	Cyclic imide	Succinimide ^a,r^	Anticonvulsant activity, antiepileptic effects, central nervous system depressant, muscle relaxant properties, sedative effects, enzyme inhibitor potential [41]
61.	Isocyanate	Aromatic isocyanate	2,6-Dimethylphenyl isocyanate ^r^	–
62.	Ketone	Diketone	1,2-Cyclopentanedione ^a^	–
63.		Acetophenone derivative	3’,5’-Dimethoxyacetophenone ^a,r^	–
64.		Cyclic diketone	4-Cyclopentene-1,3-dione ^r^	Antifungal [42]
65.		Cyclic diketone	1,2-Cyclopentanedione ^r^	–
66.		Cyclic diketone	1,2-Cyclopentanedione, 3-methyl- ^r^	–
67.		Hydroxycyclopentenone	2-Cyclopenten-1-one, 3-ethyl-2-hydroxy- ^r^	–
68.		Aryl ketone	Ethanone, 1-(1H-pyrrol-2-yl)- ^r^	–
69.		Cyclobutanone derivative	2-Dodecylcyclobutanone ^b^	–
70.	Lactone	Cyclic ester	*γ*-Butyrolactone ^a^	CNS depressant activity, sedative and hypnotic effects, anxiolytic properties, anesthetic effects, muscle relaxant, prodrug of gamma-hydroxybutyric acid (GHB), treatment of narcolepsy (via GHB), potential abuse and dependence liability [43]
71.		Furanone	2(5H)-Furanone, 3-methyl- ^r^	–
72.		Furanone derivative	2(3H)-Furanone, 5-heptyldihydro- ^r^	–
73.		Furanone	2(5H)-Furanone ^r^	–
74.		Hydroxybutyrolactone	*Aα*-Hydroxy-*γ*-butyrolactone ^r^	–
75.		Sugar lactone	(S)-(+)-2’,3’-Dideoxyribonolactone ^r^	–
76.	Nitrosoamine	Cyclic nitrosamine	N-Nitrosohexamethyleneimine ^a^	–
77.	Phenol	Methoxyphenol	Guaiacol ^a,r^	Expectorant activity, antiseptic, analgesic, anti-inflammatory, antioxidant, local anesthetic, antimicrobial [44]
78.		Allyl-substituted methoxyphenol	Eugenol ^a^	Antibacterial, antiviral, antifungal, anticancer, anti-inflammatory and antioxidant [45]
79.		Monohydroxybenzene	Phenol ^a,r^	Antiseptic, anesthetic, antibacterial, antifungal, antiparasitic, disinfectant, cauterizing agent, local analgesic [46]
80.		Alkylated methoxyphenol	Phenol, 4-ethyl-2-methoxy- ^a^	–
81.		Vinyl-substituted methoxyphenol	2-Methoxy-4-vinylphenol ^a,r^	Antioxidant, anti-inflammatory, antimicrobial, anticancer, antiplatelet, hepatoprotective, cytoprotective [47]
82.		Dimethoxyphenol	Phenol, 2,6-dimethoxy- ^a,r^	–
83.		Trihydroxybenzene derivative	5-tert-Butylpyrogallol ^a^	–
84.		Dihydroxybenzene	Hydroquinone ^a,r^	Skin depigmenting agent, antioxidant activity, anticancer potential, antibacterial activity, antifungal activity, melanin synthesis inhibition, anti-inflammatory [47,48]
85.		Hydroquinone derivative	2,3-Dimethylhydroquinone ^r^	Antioxidant activity, antimicrobial activity, cytotoxic effects, potential anticancer activity, redox-modulating [49]
86.		Pyridinol	3-Pyridinol, 6-methyl- ^r^	–
87.		Lignan derivative	4-((1E)-3-Hydroxy-1-propenyl)-2-methoxyphenol ^r^	–
88.	Pyranone	Hydroxypyranone	4H-Pyran-4-one, 2,3-dihydro-3,5-dihydroxy-6-methyl- ^r^	–
89.	Purine derivative	Heterocyclic compound	Uric acid ^r^	Potential biomarker for cardiovascular and metabolic disorders, pro-inflammatory effects in hyperuricemia, crystal-induced inflammation (e.g., gout) [50]
90.	Pyrimidine base	Nucleobase	Uracil ^r^	Antiviral, anticancer, enzyme inhibition, involvement in DNA/RNA synthesis, radiosensitizing, antimicrobial, immunomodulatory [51]
91.	Hydrocarbon	Alkyne	6-Tetradecyne ^b^	–
92.		Polyunsaturated alkene	*E*,*Z*-1,3,12-Nonadecatriene ^b^	–
93.	Terpene	Triterpene	Squalene ^b^	Anticancer, antiinflammatory, antioxidant, and antidiabetic [52]
94.		Terpenoid	Camphor ^b^	Antifungal, antiviral, and anticancer pharmacological activities, including strong inhibitory effects against *Rhizoctonia solani* and other fungal pathogens [53], antiviral activity against orthopoxviruses [54], and cytotoxic effects on various cancer cell lines via modulation of cellular pathways and structure–activity relationships [55]
95.		Terpenoid	Hedycaryol ^b^	–
96.	Phytosterol	Sterol	Chondrillasterol ^b^	Antibacterial activity against *Staphylococcus aureus* (25% inhibition), *Klebsiella pneumoniae* (38% inhibition), and *Pseudomonas aeruginosa* (65% inhibition); complete biofilm disruption of *P. aeruginosa* at 1.6 μg/mL; complete inhibition of biofilm formation at 100 μg/mL [56]
97.	Vitamin	Tocopherol antioxidant	*α*-Tocopherol ^b^	Antioxidant activity, anti-inflammatory effects, gene-regulatory activity, neuroprotective activity, cytoprotective effects, mitochondrial protection, anti-apoptotic effects, modulation of signal transduction pathways, suppression of endoplasmic reticulum stress, selective protection of non-cancerous cells during chemotherapy, attenuation of drug-induced cytotoxicity, modulation of lipid metabolism, reduction of hepatic steatosis, prevention of nonalcoholic steatohepatitis progression, interference with anti-cancer drug efficacy [57,58,59,60]
98.	Organosilicon	Silylated alkyne	1-(Trimethylsilyl)-1-propyne ^b^	–
99.	Acyl chloride	Fatty acid derivative	Palmitoyl chloride ^b^	–

Here, “a” indicates compounds identified from the aerial ethanol extract, “b” refers to compounds from the aerial oil extract, “r” represents compounds from the root ethanol extract, “a,r” represents compounds from both the aerial and root ethanol extracts, and “–“ denotes a lack of reported pharmacological activity.

**Table 6:** Common phytochemicals in ethanol extracts and major constituents of the aerial oil extract of *A. nobilis*.

Comp.	Name	Aerial Ethanol Extract	Root Ethanol Extract	Comp.	Name	Aerial Oil Extract
		Area, %	Area, %			Area, %
1	Propanoic acid	0.92	0.96	23	Camphor	1.41
2	4-hydroxybutanoic acid	7.76	2.34	24	Linoleic acid	8.08
3	Guaiacol	2.40	0.72	25	2,2,2-Trifluoro-N-(hydroxymethyl)acetamide	3.35
4	Phenol	2.01	0.61	26	Hexadecanoic acid, ethyl ester	1.15
5	2-Pyrrolidinone	3.22	0.65	27	6-Tetradecyne	14.99
6	2-Methoxy-4-vinylphenol	3.26	1.10	28	Palmitoyl chloride	2.50
7	Phenol, 2,6-dimethoxy-	2.85	1.57	29	Linoleic acid ethyl ester	1.75
8	Glycerin	15.97	26.73	30	Oleic acid ethyl ester	2.28
9	Triethylene glycol	2.80	1.05	31	*cis*-13,16-Docasadienoic acid	1.42
10	1,4:3,6-Dianhydro-*α*-d-glucopyranose	1.76	0.40	32	*α* -Tocopherol	8.36
11	Benzofuran, 2,3-dihydro-	0.60	0.41	33	*E,Z*-1,3,12-Nonadecatriene	22.25
12	Succinimide	0.48	0.34	34	Isopropyl linoleate	14.64
13	Isosorbide	7.91	0.31	35	Oleic acid, 3-hydroxypropyl ester	6.92
14	3’,5’-Dimethoxyacetophenone	2.54	9.97	36	2-Dodecylcyclobutanone	1.24
15	Tetraethylene glycol	3.22	1.95	37	Chondrillasterol	1.54
16	3-Methyl-4-phenyl-1H-pyrrole	0.61	0.48	38	Squalene	2.35
17	Hydroquinone	1.74	1.35			
18	Hexaethylene glycol	3.56	0.45			
19	Pyrrolo[1,2-a]pyrazine-1,4-dione, hexahydro-3-(2-methylpropyl)-	2.19	1.55			
20	*β*-D-Glucopyranose, 1,6-anhydro-	1.39	0.33			
21	Pyrrolo[1,2-a]pyrazine-1,4-dione, hexahydro-	1.07	0.78			
22	4-Methyleneproline	5.63	2.25			

**Table 7:** MICs of extracts against various microbial strains.

Microorganisms	Aerial Ethanol Extract, mg/mL	Aerial Oil Extract, mg/mL	Root Ethanol Extract, mg/mL
*C. albicans*	0.75	1.20	1.25
*A. fumigatus*	1.50	2.50	2.00
*C. neoformans*	0.85	1.40	1.50
MRSA	0.50	0.90	1.00
*E. coli*	1.00	1.50	1.80
*P. aeruginosa*	2.00	3.20	3.00
*K. pneumoniae*	1.20	2.10	2.00
VRE	1.00	1.60	1.80

## Data Availability

No new data were created or analyzed in this study. Data sharing is not applicable to this article.
